# Genomic ncRNAs regulating mitochondrial function in neurodegeneration: a neglected clue in the complex etiopathogenesis of multiple sclerosis

**DOI:** 10.1186/s13578-025-01438-2

**Published:** 2025-06-28

**Authors:** Nima Sanadgol, Mahedeh Samadi, Clara Voelz, Roghayeh Khalseh, Javad Amini, Cordian Beyer, Markus Kipp, Tim Clarner

**Affiliations:** 1 Institute of Neuroanatomy, RWTH University Hospital Aachen, Aachen, Germany; 2 Pharmaceutical Sciences Research Center, Faculty of Pharmacy, Tehran University of Medical Sciences, Tehran, Iran; 3 Hannover Medical School, Institute of Functional and Applied Anatomy, Hannover, Germany; 4 Natural Products and Medicinal Plants Research Center, North Khorasan University of Medical Sciences, Bojnurd, Iran; 5 Institute of Anatomy, Rostock University Medical Center, Rostock, Germany; 6 Institute of Neuroanatomy, University Hospital Bonn, Bonn, Germany

**Keywords:** Epigenetic, Demyelination, Multiple sclerosis, Mitochondria, Neuroinflammation

## Abstract

Multiple sclerosis (MS), the most prevalent myelinopathy with unclear etiology, involves mitochondrial dysfunction that critically contributes to oligodendrocyte damage and neurodegeneration. Recent interest has surged around the role of inflammatory non-coding RNAs (ncRNAs) in mitochondrial function, particularly in the context of neurodegenerative diseases (NDs), where neuroinflammation is a hallmark feature. This review highlights the collection and characterization of mitochondrial-related ncRNAs (MRncRNAs) that have been extensively studied in the context of NDs. Through a literature review, we identified 35 MRncRNAs (23 miRNAs, 8 LncRNAs, and 4 circRNAs) across Parkinson’s disease (PD), Amyotrophic Lateral Sclerosis (ALS), Alzheimer’s disease (AD), and Huntington’s disease (HD). Notably, the inflammatory miRNAs miR-34a and miR-146a were commonly dysregulated in both PD and AD, while in HD, only a single miRNA, miR-196a, was identified. As expected, due to the mitochondrial nature of PD, the majority of MRncRNAs (9 miRNAs, 8 lncRNAs, and 3 circRNAs) were associated with this disorder. Further bioinformatic analysis of MRmiRNAs revealed that miR-124-5p, -146a-3p, and -15b-3p target mitochondrial genes more than others, and mRNA of pro-apoptotic protein BCL2L11 is the most targeted. Notably, the link between these MRncRNAs and mitochondrial function in MS remains unidentified. By evaluating upregulated MS-related ncRNAs in patients and comparing them with identified MRncRNAs, we found nine overlapping miRNAs (miR-15b, miR-21, miR-27b, miR-34a, miR-124, miR-137, miR-146a, miR-155, and miR-92a) as well as two shared lncRNAs, MALAT1 and HOTAIR (called MS/MRncRNAs). Further bioinformatic analysis of MS/MRmiRNAs revealed that the autophagy pathway is the most involved. Six of these miRNAs are significantly involved in MR diseases. Notably, miR-34a-5p showed a connection to oligodendrocyte mitochondria, while miR-15b targeted two MR hub genes, SDHC and BCL2. Moreover, several hub proteins (HIF1A, STAT3, MAPK1, GSK3B) targeted by these miRNAs are well-known regulators of inflammatory pathways and mitochondrial homeostasis: These findings highlight the critical roles of ncRNAs in mitochondrial dysfunction and neurodegeneration, emphasizing the urgent need for experimental studies on MRmiRNAs, particularly in the context of MS and other myelinopathies.

**Graphical Abstract:**

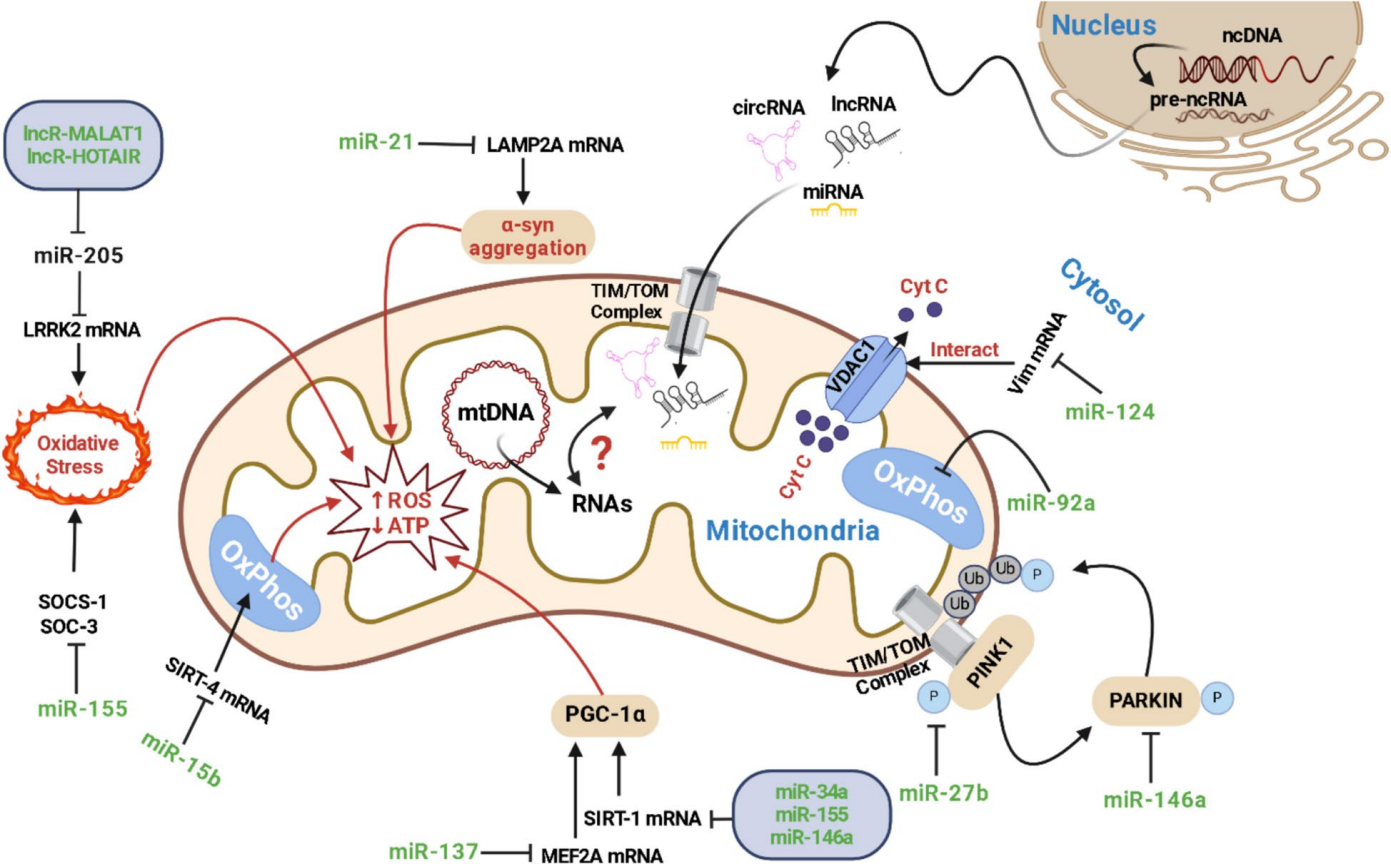

## Introduction

Neurodegenerative diseases (NDs) involve the progressive deterioration of neurons and neural tissues, leading to a variety of mental and physical impairments [13, 165]. While common mechanisms contribute to neuronal loss across different NDs, each disease's unique pathological profile is primarily driven by specific genetic mutations or the toxic aggregation of certain proteins [165]. Multiple sclerosis (MS) is a complex disease with an unknown etiology, unpredictable progression, and limited treatment options. Unlike dysmyelinating disorders (which involve abnormal myelin formation due to genetic mutations), MS involves the active destruction of previously healthy myelin by the immune system. As a prevalent myelinopathy disorder, MS manifests as a chronic, multifocal neuroinflammatory condition that leads to progressive neurodegeneration and triggers an autoimmune response (Mansour, [62, 95]). The symptomatic pathology in MS primarily arises from the infiltration of immune cells targeting the myelin sheath. Oligodendrocyte precursor cells (OPCs) attempt to differentiate and replace damaged oligodendrocytes, but various factors in the MS environment can inhibit this process [100]. This inflammatory demyelination and impaired recovery contribute to the formation of sclerotic plaques in the white matter of the brain and spinal cord. These plaques impede neurotransmission, causing permanent neurodegeneration and resulting in clinical disability [169]. In recent years, it has been demonstrated that a common factor across various neurodegenerative conditions is mitochondrial dysfunction [6, 10]. For example, mutations in genetic variants such as α-synuclein (SNCA), microtubule-associated protein tau, the glucocerebrosidase gene, Parkin (PARK2), PTEN-induced kinase 1 (PINK1), and multidrug resistance protein 1 are implicated in diseases like Alzheimer's disease (AD), Parkinson's disease (PD), and Huntington's disease (HD) [64, 78]. Evidence indicates that mitochondrial dysfunction plays a crucial role in MS, with mitochondrial damage leading to energy deficits in oligodendrocytes, thereby impairing their function and survival. Moreover, studies have shown mitochondrial abnormalities in neurons of MS patients, including changes in enzyme activity, protein levels, transcripts, and DNA structure [17, 131]. Given the crucial role of mitochondria as the primary power generators and metabolic hubs within cells, they need to be fully integrated into the cell’s regulatory and coordination systems to meet cellular demands. Consequently, ongoing communication between mitochondria and the nucleus is vital. This interaction is significantly mediated by both proteins and noncoding RNAs (ncRNAs), which work together to maintain cellular homeostasis [38]. ncRNAs, despite not coding for proteins, regulate numerous genes at the transcriptional level, thereby affecting various cellular processes (Hanieh, [7], Elham, [70]). Research has underscored their critical roles in managing genes linked to mitochondrial integrity and oxidative stress in different brain pathologies [22, 31, 91]. For example, miR-146a has been shown to influence PD by regulating parkin expression in mitochondria [63]. In this manuscript, we begin by providing a broad review of the mitochondrial-related non-coding RNAs (MRncRNAs) across a range of NDs, establishing a necessary foundation for our study. We realized that although the crosstalk between ncRNAs and mitochondria has been investigated in various NDs, especially PD, research specifically addressing this relationship in the context of MS remains limited. We then extract expression patterns of MRncRNAs across human studies to explore their potential relevance to MS. Finally, through a comparison study and bioinformatic analysis, we introduced a subset of ncRNAs that potentially contribute to MS pathology by mediating mitochondrial effects, particularly in oligodendrocytes. Given the scarcity of comprehensive studies examining this interplay in MS, and the crucial role of mitochondrial integrity in oligodendrocyte survival and brain homeostasis, our integrated approach not only contextualizes existing knowledge but also introduces a promising and underexplored avenue of MS research. Investigating how MRncRNAs may yield valuable new insights into the etiology and pathogenesis of MS.

### Mitochondrial dysfunction in MS

The etiology of MS is complex, involving a range of factors including genetic predisposition, biological factors such as obesity, immune senescence, sex hormones, and the microbiome (both gut and lung). Environmental factors such as smoking, Epstein-Barr virus infection, vitamin D levels, and stress also play significant roles [77, 119, 121]. Although no therapeutic agents can completely cure MS, current treatments can reduce relapse rates and, in some cases, delay disability progression, thereby improving patient outcomes [12, 69]. The mtDNA is particularly susceptible to oxidative damage and exhibits higher mutation rates compared to nuclear DNA (nDNA). This vulnerability is likely due to the lack of protective histones, limited DNA repair mechanisms, and heightened exposure to ROS [12, 96]. MS is categorized into three phenotypic forms: relapsing–remitting MS (RRMS), primary progressive MS (PPMS), and secondary progressive MS (SPMS). Approximately 85% of MS patients initially present with RRMS, characterized by alternating episodes of acute demyelination (relapses) and periods of neurological recovery and stability [36, 129]. A study examining the mitochondrial genome of MS patients within an Arab population, which included 23 individuals with RRMS and 24 healthy controls, identified numerous variants in the D-loop and coding regions of mtDNA. While some variants were exclusive to either the patient or control group, several common variants were found in both cohorts. Notably, the frequency of certain common variants varied between patients and controls, suggesting a potential link to MS susceptibility. Among the unique variants found only in patients, 34 were missense mutations in various mtDNA-encoded genes, reinforcing the connection between mitochondrial genetics and MS [3, 157]. Additionally, research has pointed to specific variations in the mitochondrial complex I gene as potentially associated with MS development [120]. Campbell GR et al. explored mitochondrial respiratory chain activity and mtDNA deletions in individual neurons from 13 patients with SPMS. They observed significant mtDNA deletions in neurons, including deletions in subunits of complex IV. These findings indicate that neurons in MS may suffer from respiratory deficiencies due to extensive mtDNA deletions, possibly triggered by inflammation, which may represent a key pathogenic mechanism in MS [18]. A study comparing brain tissue from 10 MS patients to that of healthy controls reported decreased activity of mitochondrial complexes I and III. Out of 119 mitochondrial electron transport chain-related genes analyzed, 26 showed significant decreases in MS samples. Mitochondrial preparations from the motor cortex of MS patients revealed a 61% reduction in complex I and 40% in complex III activity, indicating mitochondrial dysfunction and decreased ATP production in demyelinated axons. These findings suggest that reduced ATP production disrupts ion homeostasis, triggers Ca2^+^-mediated axonal degeneration, and contributes to the progressive neurological disability observed in MS [42, 151]. In parallel, Witte et al. suggested that mitochondrial changes might be a response to demyelination and inflammation. In their study of 26 MS patients, they assessed mitochondrial numbers and their co-localization with axons and astrocytes in MS lesions and normal-appearing white matter. The findings showed increased mitochondrial protein expression and a rise in mitochondrial numbers in both active and inactive lesions. Mitochondrial density was significantly higher in axons and astrocytes within active lesions compared to adjacent normal-appearing white matter, with a similar trend in inactive lesions. Additionally, complex IV activity was notably higher in MS lesions, correlating with up-regulated mitochondrial heat shock protein 70, which reflects mitochondrial involvement in the pathological processes of MS. The increase in mitochondrial density in MS lesions may contribute to free radical generation and tissue damage [161, 162]. Another study investigating mitochondrial transcriptional co-factors and proteins involved in mitochondrial redox balance in MS examined the normal-appearing grey matter from the cingulate gyrus and/or frontal cortex of 15 MS patients and 9 control subjects. The study focused on peroxisome proliferator-activated receptor gamma coactivator-1α (PGC-1α), a critical transcription factor regulating oxidative phosphorylation (OxPhos) subunits and energy metabolism, as well as mitochondrial proteins with antioxidative properties to protect neurons from ROS-induced damage. Results showed a significant reduction in PGC-1α mRNA levels in MS patients' cortical samples. This decrease was associated with neuronal loss in deep cortical layers and increased ROS production. Reduced PGC-1α expression in MS contributes to mitochondrial dysfunction and neurodegeneration, highlighting its role in the disease's progression [163]. The research also emphasizes that changes in the expression of nuclear factor erythroid 2-related factors 1 and 2 (NRF1/2), estrogen-related receptor α, and peroxisome proliferator-activated receptors can influence OxPhos gene expression, contributing to oxidative and nitrosative damage in MS. Notably, alterations in NRF-2 expression have been linked to impaired regulation of mitochondrial electron transport chain (mETC) genes and elevated ROS production in postmortem MS brains. Since NRF-2's DNA-binding activity is sensitive to redox conditions, elevated oxidative stress may impair its function and limit its binding to the promoters of mitochondrial mETC genes. This impairment may ultimately disrupt the transcription and production of mETC protein subunits, further exacerbating mitochondrial dysfunction and contributing to neurodegeneration in MS [114, 116]. Other studies investigating the mitochondrial proteome in postmortem MS and control cortex have identified significant alterations in mitochondrial protein expression. Specifically, four proteins were found to distinguish MS from controls, with three of them involved in respiration: cytochrome c oxidase subunit 5b (COX5b), the brain-specific isozyme of creatine kinase, and hemoglobin β-chain. These findings highlight the critical role of mitochondrial dysfunction in the pathogenesis of MS, further supporting the hypothesis that mitochondrial alterations contribute to the disease's progression by affecting energy metabolism and cellular function in the central nervous system (CNS) [14, 156].

## Materials and methods

### Searching methodology

In this study, we employed the MeSH terms “Mitochondria and ncRNAs,” “Mitochondria and neurodegenerative diseases,” “ncRNAs and neurodegenerative diseases,” “Mitochondria and multiple sclerosis,” “Mitochondria and Alzheimer's diseases,” “Mitochondria and Parkinson's diseases,” “Mitochondria and ALS diseases,” and “Mitochondria and Huntington diseases” to search the PubMed and Scopus databases for relevant literature up to September 2024. The review focused on original research articles written in English that explored the role of ncRNAs encoded in nuclear and mitochondrial DNA in NDs. The roles of these ncRNAs were required to be experimentally validated. Studies that solely used prediction methods to determine ncRNA targets were excluded. During the selection process, duplicate articles were removed, and the remaining articles underwent thorough evaluation. Key details extracted from each article included the type of NDs, the length and name of the ncRNA, the cellular mechanisms involving ncRNAs, and whether these mechanisms were experimentally confirmed. Articles that did not meet the inclusion criteria were excluded after a comprehensive review of their full texts. Using an alternative search strategy, we queried the same databases for all reported dysregulated ncRNAs in MS patients and collected them for future selections.

### Bioinformatic analysis of miRNAs/mRNA/protein ceRNA network

We chose miRNAs for bioinformatic analysis because their mechanisms of action and epigenetic effects are clearer and simpler compared to other ncRNAs, such as long ncRNAs (lncRNAs) and circular RNAs (circRNAs). These characteristics make it easier to evaluate and predict their potential functions. The gene targets of miRNAs were predicted using the miRDB (https://mirdb.org/) and TargetScan (https://www.targetscan.org/) databases, with common gene targets identified for each miRNA from these databases [2, 27]. Additionally, the MIENTURNET database (http://userver.bio.uniroma1.it/apps/mienturnet/) was utilized to find common target genes for a set of miRNAs [85]. Finally, only common target genes related to mitochondria were reported in this study, reflecting our focus on this organelle. The expression of miRNAs in various human brain cells was verified using miRNATissueAtlas2 (https://ccb-web.cs.uni-saarland.de/tissueatlas2) [73]. To identify direct MS-related genes targeted by miRNAs, MS-related genes were downloaded from the DisGeNET database (Disease ID: C0026769) (https://disgenet.com/) and compared with miRNA target genes [118]. Pathway enrichment analysis was performed using KEGG pathways via the Enrichr database (https://maayanlab.cloud/Enrichr/) [167]. Protein–protein interactions of miRNA target genes were verified using the STRING database (https://string-db.org/) and analyzed with Cytoscape software [149]. The RummaGEO tool (https://rummageo.com/), which mines gene expression data from the GEO database, was employed to find relationships between target genes and gene expression in diseases and cells. This tool facilitates the reuse, reanalysis, and integration of past experiments [97].

## Results

### ncRNAs and mitochondria crosstalk in ND

Despite receiving less attention than protein-coding RNAs, the study of ncRNA function has significantly advanced across multiple fields, enhancing our understanding of various biological processes [173]. While approximately 80% of the genome is transcribed into RNA, only about 2% of these transcripts encode proteins, with the majority being classified as ncRNAs [108, 173]. Numerous studies have identified ncRNAs within the mitochondria and highlighted their crucial roles in local mitochondrial protein synthesis and regulation of mitochondrial functions. While the import of cytoplasmic proteins into mitochondria is well-documented, the mechanisms facilitating the import of ncRNAs into mitochondria are less understood. It is hypothesized that a fundamental mechanism governs RNA mobilization into mitochondria, which relies on ATP and involves factors located in the mitochondrial translocase of the outer membrane (TOM). This process also depends on key components of the protein import pathway, including the TOM/translocase of the outer membrane (TIM) complexes and the voltage-dependent anion channel (VDAC) [60, 127]. The communication between mitochondria and the nucleus involves both anterograde and retrograde pathways. The anterograde pathway is where the nucleus regulates mitochondrial gene expression and function, controlling processes like mitochondrial DNA transcription and protein synthesis. In contrast, the retrograde pathway refers to mitochondrial responses to nuclear signals, regulating processes such as energy production, calcium homeostasis, and reactive oxygen species (ROS) generation. The ncRNAs play crucial roles as epigenetic regulators in these pathways. They are involved in various cellular processes, including DNA replication, chromosome maintenance, transcriptional control, RNA processing, translation, and protein transport. These functions underscore the importance of ncRNAs in maintaining cellular homeostasis and regulating mitochondrial-nuclear communication [51]. Growing research has enhanced our understanding of ncRNAs and their networks in neurodegenerative disorders, particularly through their impact on mitochondrial function. However, fully comprehending this complex interplay remains challenging [173]. In NDs, several genes have emerged as pivotal, including leucine-rich repeat kinase 2 (LRRK2), PINK1, PARK2, SNCA, and DJ-1 (PARK7). Proteins encoded by these genes are closely associated with the mitochondrial membrane, either directly or indirectly. Dysregulation of these genes leads to various mitochondrial dysfunctions, such as abnormal morphology, disrupted dynamics, increased ROS production, reduced membrane potential, impaired respiratory complex activities, and lower ATP levels [25, 28, 134].

### miRNAs and mitochondria crosstalk in NDs

miRNAs are short, single-stranded oligonucleotides, typically 19–24 nucleotides in length, that function as posttranslational regulators of gene expression [179]. They can be encoded as independent transcriptional units or located within introns and exons of protein-coding genes, and at intron–exon boundaries. miRNAs play crucial roles in cell proliferation, differentiation, development, apoptosis, cell metabolism, and the pathogenesis of various diseases [5, 103]. In neural stem cells, miR-137 has been shown to influence mitochondrial dynamics by downregulating MEF2A, thereby reducing PGC1α transcription. Interestingly, miR-137 also promotes mitochondrial biogenesis through a PGC1α-independent mechanism by upregulating NRF2 and TFAM expression, ultimately enhancing mitochondrial content and function [23]. Moreover, mitochondrial dysfunction is known to trigger the production of ROS and the release of pro-inflammatory signals such as mitochondrial DNA and cytochrome c, suggesting that certain miRNAs may play a key role in mediating neuroinflammatory responses. Among them, miR-15b has been shown to negatively regulate stress-induced SIRT4 expression. Suppression of miR-15b leads to reduced mitochondrial membrane potential and elevated ROS production through a SIRT4-dependent mechanism, indicating its role in protecting against senescence-associated mitochondrial dysfunction. Interestingly, decreased levels of miR-15b have also been reported in the peripheral blood of ALS patients. While direct evidence in ALS is lacking, the combination of these findings suggests that upregulation of miR-15b might help counteract stress-induced SIRT4 expression and potentially mitigate mitochondrial dysfunction in ALS as well [81, 86]. A comprehensive list of 35 experimentally validated MRncRNAs upregulated in NDs is provided in Table 1, while 16 downregulated ncRNAs are detailed in Table A of the supplementary material. Below is a summary of select miRNAs with particularly strong associations with mitochondrial functions.

**Table 1:** Summary of 35 experimentally confirmed upregulated ncRNAs involved in mitochondrial function/dysfunction in neurodegenerative disease

Disease	ncRNAs	Targets (translational value)	Study nature	References
PD	miR-16–1	HSP70 (Potential therapy targeting miR-16–1/HSP70 to reduce α-syn aggregation)	In vitro (SH-SY5Y cells)	[178]
	miR-21	LAMP2A (Potential therapy targeting miR-21/LAM2A to enhancing lysosomal clearance of α-synuclein)	In vitro (SH-SY5Y cells) & in vivo (mice MPTP model)	[147]
	miR-27a/b	PINK1 (Potential therapy to enhance mitochondrial clearance by modulating miR-27/PINK1 pathway)	In vitro (Human cervical HeLa and dopaminergic-like M17 cells)	[75]
	miR‑34a,b,c	PINK1 (Therapeutic potential of modulating miR-34a-5p to restore PINK1-mediated mitophagy)	In vitro (HEK293 cells) & in vivo (old mice and postmortem human brain samples)	[148]: [101])
	miR-126	Insulin/IGF-1/PI3K (Potential therapy targeting miR-126 to metabolic pathway modulation)	In vivo (postmortem human brain samples)	[76]
	miR-146a	PRKN (Targeting of miR-146a/PRKN may be protective by preserving mitochondrial function)	In vivo (rat Rotenone model)	[63]
	miR-494	DJ-1 (Potential therapeutic target by modulating miR-494/DJ-1 pathway)	In vivo (mice MPTP model)	[168]
	miR-181a	PRKN (miR-181a modulation induced mitophagy)	In vitro (SH-SY5Y cells)	[29]
	miR-7	SNCA (miR-7 induction could be neuroprotective)	In vitro (HEK293 and SH-SY5Y cells) & in vivo (mice MPTP model)	[66]
	LncRNA-NEAT1	PINK1 (NEAT1 stabilizes PINK1 and protects neurons from oxidative stress)	In vivo (mice MPTP model)	[24, 26, 142, 170]
	LncRNA-MALAT1	mirR-124, mirR-205-5p/LRRK2 (Targeting of MALAT1 as potential anti-apoptotic therapy)	In vitro (SH-SY5Y and MN9D cells) & in vivo (mice MPTP model)	[24, 26, 88]
	LncRNA-Cerox1	miR-488–3, OxPhos (Cerox1 enhances mitochondrial function and reduces oxidative stress)	In vitro (N2A cells)	[143]
	LncRNA-UCA1	P13K/Akt (UCA1 inhibition could be neuroprotective)	In vivo (rat 6-OHDA model)	[16]
	LncRNA RMST	miR-150-5p (RMST inhibition could be neuroprotective)	In vitro (SH-SY5Y cells) & In vivo (PD patients' serum)	[25, 28]
	LncRNA-SNHG1	miR-15b-5p (SNHG1 inhibition reduces α-synuclein aggregation and apoptosis)	In vitro (SH-SY5Y cells)	[24, 26]
	LncRNA-HOTAIR	LRRK2 (HOTAIR inhibition could be neuroprotective)	In vitro (SH-SY5Y cells) & in vivo (mice MPTP model)	[158]
	LncRNA-p21	miR-625/TRMP2 (lncRNA-p21 inhibition could be neuroprotective)	In vitro (SH-SY5Y and MN9D cells)	[37]
	circSNCA	miR-7/SNCA (circSNCA inhibition could reduce apoptosis and autophagy)	In vivo (mice MPTP model)	[136]
	circDLGAP4	miR-134-5p/CREB (circDLGAP4 exerts neuroprotective effects)	In vitro (SH-SY5Y cells)	[44]
	circZIP2	miR-60 (Targeting circZIP2 reduced aggregation of α-synuclein)	In vivo (*C. elegans* model)	[79]
ALS	miR-23a/b	PGC-1α (Targeting miR-23a/b could ameliorate skeletal muscle mitochondrial function)	In vitro (C2C12 cells) & In vivo (ALSG93A transgenic mice, ALS patients' skeletal muscle)	[130]
	miR-124	Vimentin (miR-124 inhibition could be neuroprotective)	In vitro (Primarily mouse motor neuron cells)	[175]
	miR-129-5p	ELAVL4/HuD (Targeting miR-129-5p could be neuroprotective)	In vivo (ALSG93A-SOD1 transgenic mice, ALS patients' peripheral blood cells)	[92]
	miR-15b	SIRT4/Cyt C (miR-15b exerts mitochondrial protection)	In vitro (primary human dermal fibroblasts) & In vivo (human blood samples)	[81, 86]
	circHDGFRP3	FUS-aggregates (HDGFRP3 localizes in stress granules in ALS-associated FUS mutations)	In vitro (HBG3 ES cells-derived motor neurons)	[35]
AD	miR-9, miR-146a, miR-34a, miR-155	SIRT1 (miRNAs reduce mitochondrial dysfunction and provide a therapeutic approach)	In vitro (SH-SY5Y and BV2 cells) & In vivo (AβO-Tg mice model, AD patients' brain)	[30]
	miR-25	KLF2, NRF2 (Potential therapeutic target by modulating miR-25/KLF2/Nrf2 axis)	In vitro (Primarily hippocampal neuron cells) & In vivo (Aβ1-42 mice model)	[41]
	miR-125b	BCL2L2/DUSP6/PPP1CA (Targeting miR-125b to prevent mitochondrial-mediated apoptosis)	In vitro (Primarily neuron cells) & In vivo (wild-type mice)	[9]
	miR-140	PINK1(Targeting miR-140 suppressed mitochondrial dysfunction, and enhanced autophagy)	In vitro (Primarily hippocampal neuron cells) & In vivo (Aβ1-42 rat model)	[83]
	miR-195	Mitofusin 2 (Targeting miR-195 suppressed mitochondrial dysfunction)	In vitro (HT22 cells) & In vivo (SAMP8 mice model)	[177]
	miR-92a	vGAT (blocking miR92a rescued the htau-induced GABAergic dysfunctions and anxiety)	In vivo (mice and postmortem human brain samples)	[84]
	miR-137	TNFAIP1 (miR-137 attenuated Aβ-induced neurotoxicity and neuroinflammation)	In vitro (primary mouse cortical neurons and N2a cells)	[59]
HD	miR-196a	CBP/PGC-1α (miR-196a exerts neuroprotective effects)	In vitro (HD-NHP cells)	[80]

*PD* Parkinson's Disease, *ALS* Amyotrophic Lateral Sclerosis, *AD* Alzheimer's, *HD* Huntington's Disease

Focusing first on PD, it was demonstrated that HSP70 is regulated by miR-16–1, and targeting the miR-16–1/HSP70 pathway is proposed as a potential therapeutic approach to reduce SNCA aggregation [178]. Moreover, elevated levels of miR-21 have been shown to directly target the 3’-untranslated region (3’-UTR) of LAMP2A (lysosome-associated membrane protein 2A), leading to a reduction in LAMP2A expression. This downregulation impairs lysosomal function and contributes to the accumulation of α-synuclein, a hallmark protein in the pathogenesis of PD [147]. In addition, miR-27a and miR-27b inhibit the translation of PINK1 by binding to its 3′-UTR. Since PINK1 is a key mitochondrial serine/threonine kinase essential for initiating mitophagy, its suppression compromises mitochondrial quality control mechanisms [75]. Similarly, miR-34a-5p has been shown to directly suppress PINK1 expression and influence mitophagy via non-canonical signaling pathways, thereby exacerbating mitochondrial dysfunction and accelerating PD progression [148]. In another study in PD, miR-34b and miR-34c were shown to regulate genes involved in mitochondrial function and oxidative stress response. Their reduced expression was associated with mitochondrial dysfunction, decreased mitochondrial complex activity, and impaired cellular viability, suggesting that miR-34b/c downregulation may contribute to early PD pathogenesis by compromising mitochondrial health [101]. It was also shown that miR-126 is involved in the regulation of the Insulin/IGF-1/PI3K pathway, and targeting miR-126 is suggested as a potential therapeutic approach for modulating metabolic pathways. This finding was observed in vivo in postmortem brains of sporadic PD patients [76]. In PD models, miR-146a has been demonstrated to downregulate Parkin protein expression, resulting in impaired mitophagy and the subsequent accumulation of damaged mitochondria, which contributes to neuronal dysfunction [63]. It has been demonstrated that miR-494 regulates DJ-1, a key protein involved in mitochondrial protection against oxidative stress, and that modulation of the miR-494/DJ-1 pathway holds potential as a therapeutic strategy for PD by preserving mitochondrial function [168]. Additionally, miR-181a has been shown to modulate PRKN (Parkin) expression, thereby promoting mitophagy, the selective clearance of damaged mitochondria, which is critical for maintaining mitochondrial quality control in PD [29]. Furthermore, miR-7 regulates SNCA expression, reducing its levels and thereby preventing mitochondrial dysfunction and exerting neuroprotective effects [66]. Moreover, cerebellar degeneration-related protein 1 antisense RNA (CDR1as) has been implicated in neuronal damage by modulating the activity of miR-7. Specifically, it downregulates miR-7, resulting in elevated levels of SNCA, a key protein involved in PD pathology [99].

The critical role of miRNAs in regulating mitochondrial function and neuroprotection in ALS has also been demonstrated by several studies. Regulation of PGC-1α, a key factor in mitochondrial biogenesis and energy metabolism, was shown to be mediated by miR-23a/b, with improvements in skeletal muscle mitochondrial function observed both in vitro and in vivo ALS models upon targeting this miRNA [130]. In motor neurons, cytoskeletal dynamics were found to be influenced by miR-124 through its regulation of vimentin, where inhibition of miR-124 was suggested to offer neuroprotection, potentially supporting mitochondrial health by stabilizing neuronal structure [175]. Furthermore, miR-129-5p was reported to target ELAVL4/HuD, an RNA-binding protein involved in neuronal survival, and modulation of this miRNA was proposed to confer neuroprotective effects in ALS models and patient samples by helping to maintain mitochondrial integrity under neurodegenerative stress [92]. Another microRNA, miR-15b, which targets SIRT4, was found to be significantly downregulated in the peripheral blood of sporadic ALS patients [86]. Previously, it had been demonstrated that miR-15b plays a protective role in mitochondrial function by preserving mitochondrial membrane potential, reducing ROS production, and modulating the expression of key mitochondrial regulatory genes such as cytochrome c, transcription factor A mitochondrial (TFAM), and nuclear respiratory factor 1 (NRF1) through the silencing of SIRT4 [81]. These findings suggest that the downregulation of miR-15b in ALS may contribute to mitochondrial dysfunction and oxidative stress, potentially exacerbating motor neuron degeneration.

In AD, several miRNAs have been identified as critical regulators of mitochondrial function, highlighting their potential as therapeutic targets. For example, miR-9, miR-146a, miR-34a, and miR-155 were shown to modulate SIRT1, thereby reducing mitochondrial dysfunction and offering neuroprotective effects in both cellular and animal models as well as in AD patient brains [30]. Additionally, miR-25 was found to influence the KLF2/NRF2 axis, a pathway essential for mitochondrial biogenesis and antioxidant responses, with modulation of this axis showing promise in hippocampal neurons and AD mouse models [41]. Targeting miR-125b was demonstrated to prevent mitochondrial-mediated apoptosis through regulation of BCL2L2, DUSP6, and PPP1CA, thereby protecting neurons from degeneration [9]. Furthermore, suppression of miR-140 enhanced mitochondrial quality control by promoting PINK1-mediated mitophagy and autophagy, as shown in hippocampal neurons and rat models of AD [83]. Lastly, miR-195 was reported to regulate Mitofusin 2, a key protein in mitochondrial fusion, with its targeting leading to improved mitochondrial function in neuronal cells and aged mouse models [177]. In another study, overexpression of full-length human tau (htau) in the mouse hippocampus was found to trigger anxiety-like behaviors by elevating miR-92a levels, which in turn downregulated vesicular GABA transporter (vGAT) expression and disrupted GABAergic signaling. Restoration of GABAergic tone, achieved through miR-92a inhibition, vGAT overexpression, or pharmacological intervention, successfully reversed both molecular impairments and behavioral abnormalities. Comparable disruptions in the miR-92a/vGAT pathway were also detected in the brains of AD patients, suggesting this regulatory axis as a promising therapeutic target [84].

In another study, miR-137 was shown to mitigate Aβ-induced neurotoxicity in Neuro2a cells by targeting Tumor Necrosis Factor Alpha-Induced Protein 1 (TNFAIP1) and inactivating the Nuclear Factor kappa-light-chain-enhancer of activated B cells (NF-κB) signaling pathway. While the primary focus was on inflammation and apoptosis, the results indirectly highlighted mitochondrial involvement, as miR-137 overexpression reduced Aβ-induced oxidative stress, preserved mitochondrial membrane potential, and inhibited mitochondrial-related apoptotic markers such as cytochrome c release and caspase-3 activation. These findings suggest a protective role of miR-137 in maintaining mitochondrial integrity under Aβ stress, potentially offering therapeutic value for Alzheimer's disease [59]. Collectively, these findings underscore the pivotal role of ncRNAs in maintaining mitochondrial integrity and suggest that modulating specific miRNAs could be a promising therapeutic strategy for alleviating mitochondrial dysfunction in AD.

In the only identified study related to Huntington’s disease, miR-196a was shown to regulate CBP (CREB-binding protein) and PGC-1α, both of which are key modulators of mitochondrial biogenesis and neuronal survival. Using an in vitro HD neural progenitor cell (HD-NHP) model, it was demonstrated that modulation of miR-196a may restore mitochondrial function and exert neuroprotective effects. These findings suggest that miR-196a could serve as a therapeutic target for mitochondrial dysfunction in HD [80]. These findings, along with the data presented in Table 1, underscore the complex roles that miRNAs play in regulating mitochondrial function and highlight their potential involvement in the pathogenesis of NDs, particularly PD.

### lncRNAs and mitochondria crosstalk in NDs

lncRNAs exceed 200 nucleotides in length and lack significant open reading frames for protein-encoding. They play crucial roles in regulating gene expression and influencing various biological processes. Their inherent stability in bodily fluids and relative ease of detection make them valuable as potential disease biomarkers. It is estimated that approximately 50% of protein-coding genes are correlated with the co-expression of a nearby lncRNA located within 50 kilobases [50]. Approximately 40% of lncRNAs are expressed in distinct neuronal tissues, where they contribute to neurobiological processes such as synaptic transmission, neural plasticity, neurogenesis, and brain development. Dysregulation of lncRNAs has been linked to the pathogenesis of various NDs [34]. Several lncRNAs have been identified as directly or indirectly influencing mitochondrial biology. They function in the cytosol by regulating mitochondrial-associated genes, often interacting with miRNAs to form a complex regulatory network for mRNA and lncRNA. Additionally, some nuclear-encoded lncRNAs are present inside mitochondria, where they exert functional effects [54].

Our findings further support the role of lncRNAs as key regulators of mitochondrial function and neuronal viability in Parkinson’s disease. Several lncRNAs have been shown to influence mitochondrial homeostasis, oxidative stress responses, and apoptotic pathways, either directly or through interactions with miRNAs and mitochondrial-related genes. A comprehensive list of eight experimentally validated upregulated lncRNAs in NDs is presented in Table 1, while details of three downregulated lncRNAs are provided in Supplementary Table A. Notably, all 11 dysregulated lncRNAs identified are associated with PD. For instance, lncRNA-NEAT1 has been demonstrated to stabilize PINK1, a key mitochondrial kinase involved in mitophagy, thereby protecting dopaminergic neurons from oxidative damage in a PD mouse model (Yan, [24, 26, 142]). lncRNA-MALAT1, through regulation of miR-124 and miR-205-5p/LRRK2, has been associated with anti-apoptotic effects and may serve as a therapeutic target to mitigate mitochondrial dysfunction [24, 26, 88]. Similarly, lncRNA-Cerox1 enhances mitochondrial OxPhos and reduces oxidative stress by modulating miR-488-3p [143]. Inhibition of lncRNA-UCA1 has shown neuroprotective effects via the PI3K/Akt pathway in a 6-OHDA rat model, suggesting its role in mitochondrial signaling [16]. Additionally, lncRNA-RMST and lncRNA-SNHG1 modulate miR-150-5p and miR-15b-5p, respectively, with their inhibition leading to decreased α-synuclein aggregation and apoptosis [24–26, 28]. Other lncRNAs such as HOTAIR and lncRNA-p21 have been shown to influence mitochondrial-related proteins including LRRK2 and TRPM2, respectively, implicating them in PD pathology through miRNA-mediated mechanisms [37, 158].

### circRNAs and mitochondria crosstalk in NDs

The circRNAs are an intriguing and unique class of endogenous RNAs that have deepened our understanding of human genetics. Unlike linear RNAs, circRNAs form a covalently closed loop by joining their 3′ and 5′ ends, which enhances their stability [25, 28, 182]. These circular molecules have been shown to serve as miRNA regulators, participate in various biological processes, and influence cellular functions through multiple mechanisms, such as miRNA sponging, protein interactions, regulation of transcription and splicing, and translation [181]. The stability of circRNAs in biological fluids, including blood, makes them promising candidates for disease biomarkers. Their expression is notably high in the brain compared to other organs, with about 20% of brain-encoded genes contributing to circRNA production. This elevated expression indicates that circRNAs may substantially influence brain function and potentially contribute to the development of neurological disorders [25, 28, 98]. It was demonstrated that the circRNA insulin-like growth factor binding protein 2 (circIGFBP2) played a key role in the development of anxiety-like behaviors following traumatic brain injury by impairing mitochondrial function and increasing oxidative stress [40]. The study showed that circIGFBP2 disrupted neural plasticity and synaptic integrity through mitochondrial dysfunction, ultimately contributing to the cognitive and behavioral deficits observed after TBI. These findings underscore the relevance of MRncRNAs in regulating neuronal resilience and stress adaptation, highlighting their therapeutic potential in neurodegenerative and neuroinflammatory disorders. Additionally, a separate functional screen identified multiple circRNAs involved in the regulation of excitatory synaptogenesis in hippocampal neurons [74]. Using loss- and gain-of-function approaches, specific circRNAs were shown to influence synaptic density and morphology, revealing their essential roles in synaptic development. This work emphasized a novel regulatory dimension through which circRNAs modulate synaptic plasticity, offering new perspectives on their contribution to brain function in both health and disease.

Our investigation identified only four circRNAs directly associated with mitochondrial function, three of which were linked to PD, and one to ALS. It was found that circSNCA was significantly downregulated by pramipexole treatment in PD models. This downregulation influenced Cell Apoptosis A through the miR-7/SNCA pathway. Specifically, reduced circSNCA levels relieved its inhibitory effect on miR-7, allowing miR-7 to suppress SNCA (alpha-synuclein) expression. As a result, pramipexole promoted Autophagy and reduced neuronal apoptosis, suggesting that circSNCA may be a critical mediator of neuroprotection in PD through its interaction with miR-7 and SNCA [136]. In another study by Feng et al., circRNA Disc Large Associated Protein 4 (circDLGAP4) was identified as a neuroprotective factor in PD models. The researchers demonstrated that circDLGAP4 acts by sponging me, thereby preventing it from downregulating CREB (cAMP response element-binding protein), a critical transcription factor involved in neuronal survival and plasticity. By modulating the miR-134-5p/CREB axis, circDLGAP4 was shown to attenuate dopaminergic neuronal damage and reduce neurodegeneration, highlighting its potential as a therapeutic target for mitigating PD progression [44]. The novel circular RNA circZIP-2, derived from the zinc finger protein 2 gene, was functionally characterized in a C. elegans model of PD. The researchers found that circZIP-2 was significantly downregulated in worms expressing human α-synuclein. This circular RNA acted as a sponge for miR-60, thereby maintaining the expression of its host gene ZIP-2. Reduced levels of circZIP-2 led to decreased ZIP-2 expression, resulting in increased α-synuclein accumulation, elevated oxidative stress, and enhanced mitochondrial dysfunction. Restoration of circZIP-2 or inhibition of miR-60 alleviated these neurotoxic effects, highlighting the circZIP-2/miR-60/ZIP-2 axis as a protective mechanism against α-synuclein-induced neurodegeneration in PD [79].

In the ALS, the circRNA derived from the Hepatoma-Derived Growth Factor-Related Protein 3 gene (circHDGFRP3) was found to shuttle along neurites and become sequestered in cytoplasmic aggregates formed by ALS-associated mutant FUS. While the primary focus of the study was on RNA localization, the disrupted trafficking of circHDGFRP3 was proposed to interfere with local RNA regulation and protein synthesis at distal neuronal sites, potentially affecting mitochondrial function. Given that mutant FUS had previously been shown to abnormally accumulate in the cytoplasm and translocate to mitochondria, leading to excessive mitochondrial fission, damage, and ultimately neuronal death, the altered localization of circHDGFRP3 was suggested to indirectly impair mitochondrial dynamics. This impairment may have contributed to the energy deficits and neurodegenerative processes characteristic of ALS pathology [35].

### ncRNAs and mitochondria crosstalk in MS

Research on MS has primarily focused on identifying specific biomarkers to improve clinical diagnosis. Given MS's classification as both a neurodegenerative and autoimmune disease and the growing recognition of ncRNAs in the pathogenesis of similar diseases, researchers are increasingly focused on identifying ncRNAs that could predict disease activity and progression [174]. Tables 2, 3, and 4 present a comprehensive list of ncRNAs with altered expression patterns in MS. However, the simple detection of these ncRNAs is insufficient; it is imperative to ascertain the signaling pathways and target genes to enhance our comprehension of their pathophysiological mechanisms. As noted earlier, extensive research has been conducted on the interaction between mitochondrial and ncRNAs in NDs, revealing that ncRNAs affect mitochondrial function [134]. In MS, while numerous studies have explored the involvement of mitochondria, research associating the expression of ncRNAs with mitochondrial function in MS is comparatively scarce when compared to other NDs.

**Table 2:** Summary of 43 upregulated miRNAs in different specimens of multiple sclerosis patients

Sample	Evaluated Groups					microRNAs	Target	Detection method	Ref
	Patients	RRMS	PPMS	SPMS	Control				
PBMC	10	10	0	0	10	miR-155, miR-92a, let-7f, miR-19a	–	Microbead, qPCR	[115]
PBMC	74	74	0	0	32	miR-142-3p, miR-155, miR-326	–	qPCR	[160]
PBMC	29	16	7	6	19	miR-21, miR-146a, miR-146b	–	qPCR	[45]
PBMC	22	22	0	0	22	let7-d, miR-744, miR-93, miR-145	–	Microarray	[145]
PBMC	50	50	0	0	50	miR-125a, miR-200c, miR-146b	–	qPCR	[172]
PBMC	40	40	0	0	20	miR-326, miR-26a	SMAD1/4	qPCR	[58]
PBMC	29	8	13	8	30	miR-155-5p, miR-223-3p	–	qPCR	[11]
PBMC, CD4^+^	43	43	0	0	42	miR-326	Ets-1	qPCR	[39]
CD4^+^/8^+^	23	23	0	0	10	miR-17-5p	PI3KR1, PTEN	qPCR	[87]
Treg	22	22	0	0	24	miR-19b	TGFβ	Microarray	[33]
PBMC, CD4^+^	22	12	5	5	16	miR-128, miR-27b, miR-340	BMI1 GATA3, IL-4	qPCR	[53]
PBMC, CD4^+^	19	11	4	4	17	miR-29b	IFN-γ	qPCR	[144]
CD4^+^/8^+^	24	5	1	18	9	miR-16, miR-155, miR-142-3p	FOXP3, PDCD1, IRF2BP2, FOXO1	qPCR	[4]
PBMC, B cells	14	14	0	0	13	miR-132	Sirtuin-1	qPCR	[102]
PBMC	-	-	-	-	-	miR-155, miR-146a	–	qPCR	[105]
PBMC, CD4^+^	20	20	0	0	20	miR-34a, miR-30c, miR-19a	–	qPCR	[48]
Whole blood	20	20	0	0	19	Let-7c, miR-233	–	Microarray	[71]
Whole blood	5	5	0	0	21	miR-22-5p, miR-27a-5, miR-467	–	NGS, Microarray, qPCR	[72]
Whole blood	34	34	0	0	16	miR-223	ARG1, STAT3	qPCR	[19]
Plasma/serum	4	4	0	0	4	miR-614; miR-572; miR-648; miR-422a; miR-1826; miR-22	–	Microarray	[141]
Plasma/serum	62	62	0	0	55	miR-145	–	qPCR	[145]
Plasma/serum	31	31	0	0	31	miR-155; miR-326; miR-146a; miR-150; miR-124	–	qPCR	[176]
Plasma/serum	62	0	31	31	21	miR-128-3p; miR-376c-3p; miR-26a-5p; miR-191-5p	–	qPCR	[154]
Plasma/serum	37	18	0	19	23	miR-145; miR-223	–	qPCR	[140]
Plasma/serum	84	36	20	28	50	miR-320a; miR-486-5p; miR-320b; miR-25-3p; miR-140-3p; let-7 c-5p	–	qPCR	[124]
Plasma/serum	73	53	20	0	27	miR-24-3p; miR-191-5p	–	qPCR	[155]
Frontal white matter	6	1	1	4	6	miR-142-5p	SOCS1, TGFBR-1	qPCR	[150]
Brain lesion (Astrocytes)	16	–	–	–	9	miR-155, miR-34a, miR-326	CD47	qPCR	[67]
Neurovascular Unit	6	–	–	–	6	miR-146a	NFκB	qPCR	[164]
Neurovascular Unit	6	1	1	4	6	miR-155	Annexin-2, Claudin-1, DOCK-1, Syntenin-1	qPCR	[93]

*PBMC* Peripheral blood mononuclear cells

**Table 3:** Summary of 33 differentially expressed LncRNAs in different specimens of multiple sclerosis patients

Sample	Evaluated Groups					LncRNA (expression)	Target	Detection method	Ref
	Patients	RRMS	PPMS	SPMS	Control				
PBMC	12	–	–	–	15	LINC00649 (↑), MALAT1, P73-AS1	B cell receptor	qPCR	[159]
Whole blood	38	38	0	0	37	LNC-MKI67IP (↓), HNF1A-AS1 (↓), LINC00305(↓)	NF-κB pathways	qPCR	[132]
Whole blood	50	–	–	–	50	NORAD (↑), RAD51-AS1 (↑), ZNRD1ASP (↓)	MAPK	qPCR	[49]
Choroid plexus	6	6	0	0	6	HIF1A-AS3 (↑), SNHG1 (↑)	ND	RNA-seq, qPCR	[128]
PBMC	51	–	–	–	51	THRIL (↑)	ND	qPCR	[113]
Whole blood	50	50	0	0	50	PINK1-AS (↑)	ND	qPCR	[117]
Whole blood	50	50	0	0	50	RMRP (↑), FLICR (↑)	ND	qPCR	[32]
Serum	118	61	22	35	20	BACE1-AS (↑), BC200 (↑)	ND	qPCR	[68]
Serum	45	45	0	0	45	MALAT1 (↑), Inc-DC (↑)	ND	qPCR	[139]
Serum	28	0	12	16	8	LINC00293 (↑), RP11-29G8.3 (↑), TUG1 (↑)	ND	qPCR	[138]
Serum	12	12	0	0	12	NEAT1 (↑), TUG1 (↑), RN7SKRNA (↑)	ND	qPCR	[137]
PBMC	83	83	0	0	44	RORC (↑), DDX5 (↑), RMRP (↑)	ND	qPCR	[123]
PBMC	50	50	0	0	25	RP11-530C5.1 (↑), AL928742.12 (↓)	ND	qPCR	[50]
Serum	120	60	0	30	30	RUNXOR (↓)	RUNX1	qPCR	[56]
Whole blood	50	50	0	0	50	lnc-DC (↑)	ND	qPCR	[8]
Whole blood	20	20	0	0	10	NR_003531.3 (↓)	ND	qPCR	[106]
Whole blood	40	40	0	0	40	SPRY4-IT1 (↓), HOXA-AS2 (↓), LINC-ROR (↓), MEG3 (↓)	ND	qPCR	[133]
PBMC	42	42	0	0	32	HOTAIR (↑)	ND	qPCR	[112]

*PBMC* Peripheral blood mononuclear cells

**Table 4:** Summary of 15 differentially expressed circRNAs in different specimens of multiple sclerosis patients

Sample	Evaluated Groups					circRNA (expression ↑↓)	Target	Detection method	Ref
	Patients	RRMS	PPMS	SPMS	Control				
PBMC	67	67	0	0	37	circRNA_101348 (↑), circRNA_102611 (↑), circRNA_104361 (↑)	–	Microarray	[183]
PBMC	65	65	0	0	37	circRNA_001896 (↓), circRNA_101145 (↓)	–	Microarray, qPCR	[107]
PBMC	10	10	0	0	10	circ_0007990 (↓)	–	RNA-Seq	[21]
CD4^+^	18	18	0	0	20	circINPP4B (↑)	–	Microarray	[55]
PBMC	30	30	0	0	30	hsa_circ_0106803 (↑)	–	qPCR	[20]
CSF, Whole blood	56	20	17	19	20	circ_0000518 (↑)	FUS, CaMKKβ, AMPK	qPCR	[65]
Leukocytes	30	20	0	10	20	hsa_circ_0141241 (↑), hsa_circ_0058514 (↑), hsa_circ_0001947 (↑), hsa_circ_0001707 (↑), hsa_circ_0001400 (↑), hsa_circ_0001459 (↑)	–	RNA-Seq	[61]

#### miRNAs and mitochondria crosstalk in MS remain underexplored

In 2009, the initial exploration of miRNA expression profiles in patients with MS marked a pivotal milestone [111]. Subsequently, several studies in the field of MS have investigated miRNA expression profiles in various biological samples, including whole blood, brain lesions, peripheral blood-derived lymphocytes, and serum samples. These studies have yielded diverse expression profiles of numerous miRNAs, shedding light on the complex landscape of miRNA involvement in MS pathogenesis [5]. In Table 2, certain studies have identified the targets of microRNAs, while others have solely reported the changes in their expression. Furthermore, some studies illustrate interactions between miRNA expression and genes potentially affecting mitochondrial function. Details on the downregulated miRNAs can be found in Supplementary Table B. Although these studies do not directly investigate mitochondria, they demonstrate the potential for investigating the role of ncRNAs in mitochondrial functions within the context of MS disease. One study found that the overexpression of miR-132 led to the suppression of SIRT1. This suppression was linked to abnormal increases in LT and TNFα production by MS B cells. SIRT1 is involved in various physiological and pathological processes, including mitochondrial biogenesis via PGC1-alpha [102, 134]. Another study observed an inverse relationship between the downregulation of the miR-15a and miR-16–1 cluster and the overexpression of the B-cell lymphoma 2 (BCL2) gene in CD4^+^ T-cells from MS patients. This finding highlights the potential role of these miRNAs in MS pathophysiology. The BCL-2 gene is crucial for regulating cell survival and apoptosis by controlling mitochondrial membrane integrity and the release of pro-apoptotic molecules [94]. Additionally, increased expression of miR-142 isoforms in the CNS of MS patients was noted. These miRNAs contribute to autoimmune neuroinflammation by targeting and suppressing protective genes such as SOCS1, which mitigates oxidative stress and protects mitochondria [150].

#### LncRNAs and mitochondria crosstalk in MS remain underexplored

In recent years, numerous studies have examined the role of lncRNAs in MS disease. Among the array of biomarkers examined in MS, LncRNAs hold significant importance due to their capacity to regulate genome expression and stability and influence cellular functions including proliferation, differentiation, apoptosis, and development. Table 3 presents a list of dysregulated lncRNAs in MS. Based on the findings of these studies and considering the capacity of lncRNAs to modulate gene expression through diverse mechanisms at various levels, they play a pivotal role in the development of MS. [5, 109]. For instance, Hafouri-Fard et al. reported that two MAPK14 (Mitogen-activated protein kinase 14)-related lncRNAs, NORAD and RAD51-AS1, were up-regulated. MAPK14, a protein kinase induced by cell stress and inflammatory stimuli, plays a significant role in inflammation and cell death, making it a potential target for MS therapeutic interventions [49]. Several nuclear-encoded lncRNAs have been identified within mitochondria, suggesting a potential role in regulating mitochondrial functions. These lncRNAs may influence various aspects of mitochondrial function by directly targeting or indirectly affecting mitochondrial-related genes, transcripts, or proteins [54]. For example, our previous findings demonstrate a significant link between redox imbalance and the dysregulation of mitochondrial LncRNA growth arrest-specific 5 and miR-651-5p expression in the HMC3 cell line. Notably, the increased expression of miR-651-5p in exosomes under stress conditions suggests that the transport of miR-651-5p into serum exosomes may vary and be associated with GAS5 expression in PBMCs across different MS subtypes [104]. However, as mentioned above in the context of miRNA, to date, there are no specific studies investigating the relationship between lncRNA expression and mitochondria in the context of MS.

#### circRNAs and mitochondria crosstalk in MS remain underexplored

Several studies support the hypothesis that circRNAs could serve as potential biomarkers for NDs. However, one of the latest challenges in ncRNA research involves the study of circRNAs [31]. These studies are summarized in Table 4. Although circRNAs have recently been implicated in MS pathogenesis, their role remains largely unexplored, highlighting the need for further investigation into the molecular pathways they may influence. Moreover, this emerging field could become a key focus for exploring the unique molecular mechanisms governing mitochondrial gene expression, extending beyond MS to include other NDs.

### MS-associated ncRNAs that their mitochondria function confirmed in other NDs

**Fig. 1:**
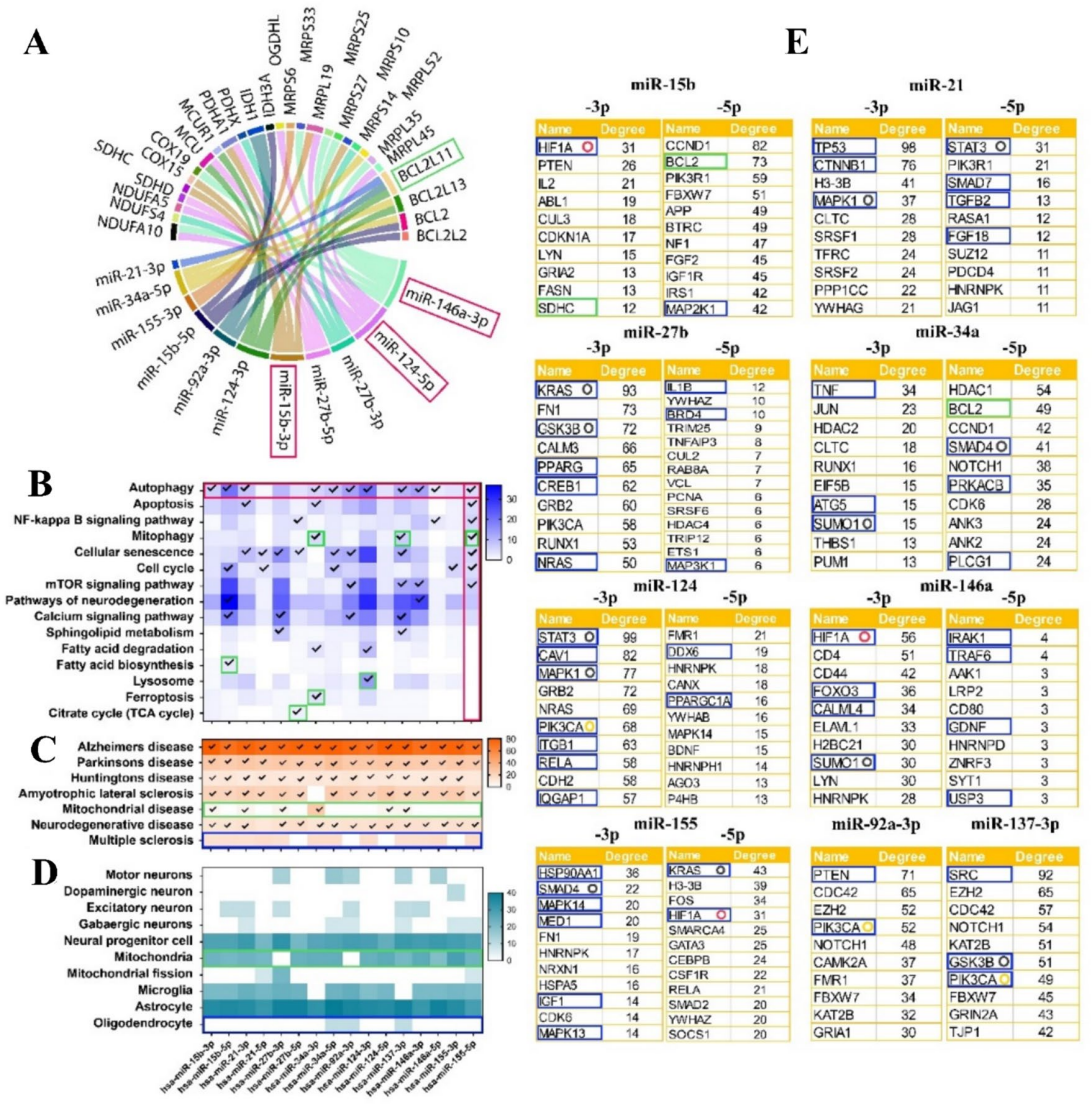
Analysis of Common miRNA Targets from Table 1, Focusing on Mitochondrial Genes. **A** Identification of mitochondrial genes frequently targeted by the listed miRNAs. Our results show that 11 miRNAs target at least one mitochondrial gene, with miR-124-5p, miR-146a-3p, and miR-15b-3p targeting the highest number. BCL2L11, a pro-apoptotic protein, was the most targeted mitochondrial gene, identified as a target of three miRNAs. *Bioinformatic analysis of 16 miRNAs (both -3p and -5p arms) from *Table 5*.* **B** Pathway analysis reveals that 16 miRNAs predominantly regulate the autophagy pathway (11 miRNAs, horizontal red rectangle), with miR-155-5p involved in the highest number of pathways (7 pathways, vertical red rectangle). Mitochondrial-related pathways, such as mitophagy, fatty acid biosynthesis, lysosome, ferroptosis, and the citrate cycle, are targeted by at least one miRNA (5 miRNAs, green square). Additionally, calcium signaling is targeted by four different miRNAs. **C** Disease association analysis indicates that nearly all selected miRNAs are implicated in neurodegenerative diseases (PD, ALS, AD, HD), with six linked to mitochondrial-related diseases (horizontal green rectangle). However, none have been reported concerning MS pathogenesis (horizontal red rectangle). **D** Cell-specific expression analysis shows that these miRNAs are highly expressed in neural progenitor cells, microglia, and astrocytes, with significant links to mitochondrial function (horizontal green rectangle). In contrast, fewer reports indicate their expression in dopaminergic neurons (linked to mitochondrial fusion) or oligodendrocytes (horizontal blue rectangle). **E** Protein–protein interaction (PPI) analysis of target mRNAs highlights key hub genes. miR-15b (-3p/-5p) and miR-34a-5p specifically target mitochondrial proteins such as succinate dehydrogenase complex subunit C (SDHC) and BCL2, which are essential for mitochondrial function (green rectangles). Hypoxia-inducible Factor 1 Alpha (HIF1A) and Phosphatidylinositol-4,5-Bisphosphate 3-Kinase Catalytic Subunit Alpha (PIK3CA) are identified as hub proteins targeted by three miRNAs (red and orange circles). Most miRNAs also target several mitochondrial-related proteins (blue rectangles). Additional hub proteins targeted by at least two miRNAs include Mitogen-Activated Protein Kinase 1 (MAPK1), Signal Transducer and Activator of Transcription 3 (STAT3)**,** Glycogen Synthase Kinase 3 Beta (GSK3B)**,** KRAS Proto-Oncogene, GTPase (KRAS)**,** SMAD Family Member 4 (SMAD4)**,** and Small Ubiquitin-Like Modifier 1 (SUMO1) (black circles)

Table 5 compiles ncRNAs appearing in other tables of this manuscript to identify those investigated in both MS and potentially influencing NDs via mitochondrial effects. While these ncRNAs' roles in NDs have been confirmed through their impact on mitochondrial function (Table 1), their involvement in MS is less explored. Our analysis identified 9 miRNAs (miR-15b, miR-21, miR-27b, miR-34a, miR-124, miR-137, miR-146a, miR-155, and miR-92a) and 2 lncRNAs (MALAT1 and HOTAIR) associated with MS (Table 5 and graphic abstract). Using data from Tables 1 and 5, we conducted a bioinformatic analysis to better understand the relationship between the identified miRNAs. Initially, we evaluated common targets of miRNAs from Table 1, focusing exclusively on mitochondrial genes. This allowed us to identify miRNAs with the most targets among these genes and the mitochondrial genes most frequently targeted by these miRNAs (Fig. 1A). Our results revealed that 11 miRNAs target at least one mitochondrial gene, with miR-124-5p, miR-146a-3p, and miR-15b-3p targeting higher mitochondrial genes (Fig. 1A). Additionally, BCL2L11, a pro-apoptotic protein, emerged as the most targeted mitochondrial gene, being targeted by three MRncRNs (Fig. 1A). We further analyzed the 16 miRNAs (both arms -3p and -5p) from Table 5 using bioinformatic tools, presenting the findings in Figs. 1B–E. This analysis showed that 16 different miRNAs predominantly involve the autophagy pathway (11 miRNAs, highlighted by the horizontal red rectangle), and miR-155-5p is involved in more pathways (7 pathways, highlighted by the vertical red rectangle) (Fig. 1B). Notably, mitochondrial-related pathways, including mitophagy, fatty acid biosynthesis, lysosome, ferroptosis, and the citrate cycle, are each targeted by at least one miRNA (5 miRNAs, highlighted by the green square), while Ca signaling is targeted by four different miRNAs (Fig. 1B). Our evaluation of the roles of these selected miRNAs in human disease development confirmed our previous findings, revealing that nearly all of them are involved in the pathogenesis of PD, ALS, ALZ, HD, and other NDs (Fig. 1C). Additionally, six of these miRNAs are implicated in mitochondrial-related diseases (highlighted by the horizontal green rectangle), while none have been reported concerning the pathogenesis of MS (highlighted by the horizontal red rectangle) (Fig. 1C). Subsequent analysis of their cell-specific expression demonstrated high expression levels in neural progenitor cells, microglia, and astrocytes, with significant reports linking them to mitochondrial function (highlighted by the horizontal green rectangle) (Fig. 1D). Interestingly, there are fewer reports of these miRNAs being expressed in dopaminergic neurons, involved in mitochondrial fusion, or expressed in oligodendrocytes (highlighted by the horizontal blue rectangle) (Fig. 1D). Finally, we performed a protein–protein interaction analysis on the target mRNAs of each selected miRNA, highlighting the top hub genes presented in Fig. 1E. The analysis revealed that miR-15b (-3p and -5p) and miR-34a-5p have specific mitochondrial proteins among their hub proteins, such as succinate dehydrogenase complex subunit C (SDHC) and BCL2, which are critical for mitochondrial function and cellular fate (highlighted by the green rectangle) (Fig. 1E). Additionally, Hypoxia-Inducible Factor 1 Alpha (HIF1A) and Phosphatidylinositol-4,5-Bisphosphate 3-Kinase Catalytic Subunit Alpha (PIK3CA) were identified as hub proteins in the target networks of three different miRNAs (highlighted by the red and orange circles), and nearly all other miRNAs had several mitochondrial-related proteins among their hub proteins (highlighted by the blue rectangles) (Fig. 1E). Other hub proteins targeted by at least two miRNAs included MAPK1, STAT3, GSK3B, KRAS, SMAD4, and SUMO1 (highlighted by the black circles) (Fig. 1E). Our various bioinformatic analyses of this group of miRNAs confirmed their extensive association with mitochondria, suggesting they may play a critical role in the pathogenesis of MS.

**Table 5:** Shared ncRNAs in multiple sclerosis and other neurodegenerative diseases, specifically focusing on mitochondrial functions

microRNA	Expression in MS (↑↓)	Expression in other NDs (↑↓)	Mitochondria-related function
miR-15b	↓[46, 126]	ALS-↓[81, 86]	Downregulation leads to mitochondrial dysfunction and redox homeostasis by impacting SIRT4 expression [81, 86]
miR-21	↑[126]	PD-↑[147]	Over-expression leads to the downregulation of LAMP2A, potentially contributing to increased levels of α-synuclein protein [147]
miR-27b	↑[53]	PD-↑[75]	Upregulation leads to mitochondrial dysfunction by suppressing PINK1 expression [75]
miR-34a	↑[48]	AD-↑[30]	Downregulates SIRT1 by binding to the 3′ UTR of SIRT1 mRNA. This suggests a potential mechanistic connection between BDNF expression and mitochondrial biogenesis[30]
miR-124	↑[176]	ALS-↑[152]	Targets Vim and regulates mitochondrial function and localization [152]
miR-137	↓[43]	AD-↓[110]	MEF2A is a downstream target of miR-137. Decreased levels of MEF2A lead to reduced transcription of PGC1α, consequently impacting mitochondrial dynamics [110]
miR-146a	↑[1, 45]	PD-↑[15, 63]; AD-↑[30]	Accumulates the damaged and dysfunctional mitochondria through the reduction of the levels of Parkin protein [63]. miR-146a affects mitochondrial function through the regulation of the SIRT1 protein level[30]
miR-155	↑[93]	PD-↑[15]; AD-↑[30]	Suppresses SOCS-1 and SOCS-3 protein expression and leads to mitochondrial dysfunction [15]. miRNA-155 targets the SIRT1 mRNA 3′UTR, downregulates SIRT1 expression, leading to mitochondrial dysfunction [30]
LncRNA-MALAT1	↑[139]	PD-↑[88]	The upregulation of LRRK2 expression resulting from the inhibition of miR-205 through MALAT1 leads to mitochondrial dysfunction and apoptosis [88]
LncRNA-HOTAIR	↑[112]	PD-↑[158]	The upregulation of LRRK2 expression resulting from the inhibition of miR-205 through HOTAIR leads to mitochondrial dysfunction and apoptosis [158]

### Mitochondrial-genome encoded ncRNAs

The above-mentioned research has identified ncRNAs derived from the nuclear genome that influence mitochondrial function. There is an additional category of ncRNAs encoded directly within mitochondrial DNA. Rackham et al. identified mtDNA-encoded non-coding RNAs, comprising approximately 14% of the mitochondrial transcriptome, excluding rRNAs and tRNAs [122]. Therefore, in addition to encoding transcripts for oxidative phosphorylation, the human mitochondrial genome harbors a diverse array of ncRNA transcripts that play crucial biological roles within the cell (Nima, [135]). Several research studies have pinpointed numerous ncRNAs transcribed by the human mitochondrial genome, suggesting potential involvement in regulating mitochondrial gene expression, safeguarding mitochondrial DNA from damage, and acting as precursors for other categories of ncRNAs [108, 125].

Three lncRNAs originating from the mitochondrial genome—lncND5, lncND6, and lncCytB—have been identified within mitochondria [122]. Their expression is regulated by nuclear-encoded mitochondrial processing proteins, particularly the mitochondrial RNase P (MRP) complex. The mitochondrial ribonuclease P protein 1 (MRPP1) and mitochondrial ribonuclease P protein 3 (MRPP3) influence the overall abundance of these lncRNAs, while pentatricopeptide repeat-containing protein 2 (PTCD2) specifically modulates the processing of lncND5. Among them, lncND5 is the most abundant, though its expression varies across tissues, and all three contribute to mitochondrial gene expression regulation [122]. Additionally, several chimeric lncRNAs incorporating segments of mitochondrial DNA (mtDNA)-encoded genes have been identified. The first to be discovered, sense noncoding mitochondrial RNA (SncmtRNA), comprises mitochondrial 16S rRNA fused with a 121-nucleotide and an 815-nucleotide 5′-leader sequence from the complementary strand, forming an RNase-resistant double-stranded structure with a 40-nucleotide loop [153]. Since then, two additional 16S rRNA chimeric lncRNAs have been reported: antisense noncoding mitochondrial RNA 1 (ASncmtRNA-1), which includes a 310-nucleotide 5′-leader sequence from the antisense strand, and ASncmtRNA-2, a stem-loop structure linking the short arm of the 5′ end of the 16S rRNA to its full antisense sequence [171]. Notably, ASncmtRNA-1 and ASncmtRNA-2 are found in both mitochondria and the cell nucleus, suggesting a potential role in retrograde signaling [82].

Studies have shown that several miRNAs align with the mitochondrial genome at sites corresponding to 16S rRNA, tRNA, and mRNA, potentially influencing the turnover of target mRNAs. However, it remains unclear whether these miRNAs are transcribed from the mitochondrial genome [146]. Moreover, mitochondrial genome-encoded circRNAs in mammals remained unidentified until recent studies reported the discovery of hundreds of mitochondria-encoded circRNAs (mecciRNAs) in human and mouse cells using second-generation mitochondrial RNA sequencing [90]. Interestingly, some mecciRNAs localize both within mitochondria and in the cytosol [89]. Functional studies on mecciND1 and mecciND5 revealed their essential roles. MecciND1, encoded by the ND1 gene, binds RPA1 and RPA2, proteins involved in mtDNA replication, with its expression positively correlating with mtDNA copy numbers. MecciND5, from the ND5 gene, interacts with hnRNPA1, hnRNPA2B1, and hnRNPA3, aiding their mitochondrial import [89]. Both mecciRNAs associate with TOM40 and PNPASE, acting as molecular chaperones for nuclear-encoded polypeptide import. They are upregulated in hepatocellular carcinoma and regulated under stress, highlighting their role in cellular adaptation [89].

Moreover, Zhao et al. found that three mitochondria-encoded circRNAs were downregulated in liver fibroblasts from NASH patients [180]. One, SCAR (Steatohepatitis-associated circRNA ATP5B Regulator), an antisense RNA from cytochrome c oxidase subunit 2 (COX2), inhibited mROS production by interacting with ATP5B, potentially serving as a therapeutic target. Another, mc-COX2 (a sense RNA from the locus COX2), highly expressed in CLL patient plasma exosomes, was linked to prognosis, promoting cell proliferation and inhibiting apoptosis [166]. These ncRNAs have not been studied concerning ND and represent novel and compelling topics that warrant further investigation.

#### Role of mitochondria epigenome in MS remains underexplored

Mitochondria are intricate organelles with two membranes that contain their DNA and can replicate independently of the host cell. These dynamic structures are essential for generating the majority of cellular energy through OxPhos [57]. Effective OxPhos and ATP production are critical for cellular homeostasis and survival. Beyond ATP synthesis, mitochondria also play vital roles in redox regulation, calcium (Ca^2+^) storage, essential compound metabolism, and apoptosis [47]. The replication, transcription, and translation of mitochondrial DNA (mtDNA) are managed by a specialized non-coding region known as the displacement loop (D-loop). Each cell harbors numerous mtDNA copies, ranging from several hundred to tens of thousands, depending on the cell type. The mitochondrial genome consists of 37 genes, including 13 that code for subunits of the respiratory chain. Additionally, it includes 22 transfer RNAs and 2 ribosomal RNAs (12S and 16S rRNAs), which are essential for mitochondrial translation [52, 78]**.** Mitochondrial-encoded ncRNAs serve as mediators of communication between the mitochondria and the nuclear genome, primarily through retrograde signaling, a process in which the mitochondria transmit ncRNAs to convey environmental stress signals, leading to changes in nuclear gene expression [52]. This mechanism has implications for stress responses, including potential contributions to disease. More than 31 distinct ncRNAs have been identified in mammals, originating from the mitochondrial genome [125]. Additionally, ncRNAs are involved in biological roles such as the import of proteins into mitochondria and the regulation of mitochondria function [60, 127]. Moreover, nuclear DNA shares significant homology with mtDNA, and mitochondrial pseudogenes within the nuclear genome are referred to as nuclear mitochondrial DNA sequences (NUMTs). This homology can complicate the determination of the genome of origin. While some studies have demonstrated that small mitochondrial RNAs are transcribed by the mitochondrial genome rather than by NUMTs through various methods, the potential for NUMTs to produce ncRNAs remains an intriguing and challenging question [10]. Given the CNS dependence on mitochondrial energy production and the critical role mitochondria play in cellular functions, mitochondrial epigenome dysfunction may be implicated in developing various diseases, including MS (Nima, [135].

## Conclusions and perspectives

Emerging studies suggest that ncRNAs are pivotal in orchestrating essential biological processes within cellular and mitochondrial contexts. Specifically, the interaction between ncRNAs and mitochondria has attracted considerable attention from researchers focused on NDs. However, despite the wealth of research on the expression, mechanisms, and characteristics of ncRNAs in MS, there is a marked lack of studies exploring the specific interactions between ncRNAs and mitochondrial functions. A growing body of evidence highlights mitochondrial dysfunction, driven by a complex interplay of genetic and epigenetic factors, as a critical aspect of MS pathology. To bridge this gap, we have identified and compiled a shortlist of ncRNAs that warrant comprehensive investigation within the context of MS, particularly concerning their roles in mitochondrial pathways. Mitochondrial function is crucial for oligodendrocyte biology, particularly in the context of neuroinflammatory diseases such as MS. Mitochondria provide the necessary ATP for myelin synthesis and maintenance, and their dysfunction can lead to impaired energy production, contributing to myelin degradation. Additionally, mitochondria regulate ROS levels, and excessive ROS can cause oxidative stress, damaging oligodendrocytes and myelin. Mitochondria also play a role in apoptosis regulation, and their dysfunction can trigger oligodendrocyte apoptosis, exacerbating demyelination. Furthermore, proper mitochondrial dynamics are essential for cellular health, and any imbalance in fusion and fission processes can impair oligodendrocyte function. Thus, mitochondrial dysfunction in oligodendrocytes is a significant factor in the pathogenesis of MS, highlighting the need for therapeutic strategies that protect and support mitochondrial health to combat demyelination and promote remyelination. Due to the difficulty of accessing human brain tissue, most of the ncRNAs identified in our investigation are derived from blood samples and proposed as potential biomarkers without further analysis of their specific donor cells in the brain. This leaves a significant gap in understanding whether their dysregulation in circulation contributes to MS etiology or results from the disease's pathological conditions. Although numerous ncRNAs have been linked to MS through microarray or RNA-seq studies, a key limitation is the frequent lack of functional validation. These methods often generate descriptive lists of dysregulated circRNAs without confirming their biological relevance. Without follow-up experiments, such as qPCR, RNase R treatment, or gain- and loss-of-function assays, the role of these ncRNAs in MS remains unclear. This limits our understanding of their mechanistic contribution and hinders their development as therapeutic targets or biomarkers. Future studies must pair transcriptomic data with rigorous functional characterization. To address this limitation, we conducted a bioinformatic analysis using available databases to identify potential epigenetic mechanisms mediated by these miRNAs, with a specific focus on their impact on mitochondrial function. Our bioinformatic analyses provide key insights into the relationship between specific miRNAs and mitochondrial function, potentially implicated in MS pathogenesis. For instance, we identified 11 miRNAs that target mitochondrial genes, with miR-124-5p, miR-146a-3p, and miR-15b-3p being particularly prominent. Notably, BCL2L11, targeted by three miRNAs, emerged as a critical mitochondrial gene. BCL2L11 is a pro-apoptotic member of the Bcl-2 protein family and plays a crucial role in initiating apoptosis by binding to and antagonizing the anti-apoptotic proteins Bcl-2 and Bcl-xL. Lower levels of BCL2L11 due to miRNA targeting can lead to decreased apoptosis, promoting cell survival. Further analysis revealed that differentially expressed miRNAs common to both NDs and MS are mainly involved in the autophagy pathway, and are less expressed in oligodendrocytes. Overall, integrating these miRNA data suggests that the absence of anti-apoptotic miRNAs, coupled with reduced autophagy, may contribute to significant oligodendrocyte loss and neuroinflammation in MS. In contrast, PP interaction analysis highlighted miR-15b and miR-34a-5p targeting essential mitochondrial hub proteins such as SDHC and BCL2. SDHC is a critical component of the mitochondrial respiratory chain, specifically complex II, which is involved in both the electron transport chain and the citric acid cycle. Reduced SDHC expression impairs electron transport and ATP production, leading to decreased cellular energy levels and increased production of ROS. Concurrently, BCL2 is an anti-apoptotic protein that helps maintain mitochondrial membrane integrity and prevents the release of cytochrome c, a key step in the apoptotic pathway. Lower levels of BCL2 increase the susceptibility of cells to apoptosis due to diminished protection against pro-apoptotic signals. In the context of oligodendrocytes, such mitochondrial dysfunction and heightened apoptosis can contribute to myelinopathy, exacerbating conditions like MS. Thus, the decreased expression of SDHC and BCL2 disrupts mitochondrial function, energy production, and cell survival, potentially leading to significant neurodegenerative consequences.

Finally, HIF1A and PIK3CA emerged as common hub proteins among several miRNAs, with other significant targets including MAPK1, STAT3, GSK3B, KRAS, SMAD4, and SUMO1 (Fig. 1E). When HIF1A and PIK3CA are downregulated in oligodendrocytes, it can lead to significant disruptions in their function and survival, impacting the myelination process. HIF1A is crucial for cellular responses to hypoxia. Downregulation of HIF1A in oligodendrocytes can impair their ability to adapt to hypoxic stress, reducing their viability and function. PIK3CA, part of the PI3K/AKT signaling pathway, plays a pivotal role in cell growth, survival, and metabolism. PIK3CA activation supports cell survival, differentiation, and myelin production in oligodendrocytes. Downregulation of PIK3CA disrupts these processes, leading to increased apoptosis and reduced myelination. In summary, downregulation of HIF1A and PIK3CA in oligodendrocytes undermines their ability to cope with hypoxic conditions, reduces cell survival and myelination capacity, and impairs the repair of myelin damage, contributing to the progression of demyelinating diseases. It should be noted that our study showed that there is limited data on these miRNAs concerning MS and their expression in oligodendrocytes, underscoring the need for further evaluations in this area. Understanding the crosstalk between ncRNAs and mitochondria in MS is an intriguing and underexplored area of research that raises numerous compelling questions with potential clinical significance. This exploration offers the potential to uncover novel therapeutic targets and approaches, which could significantly enhance the treatment and management of MS. Continued investigation in this field is essential for advancing our understanding and developing new interventions that address the complex pathology of MS.

## Data Availability

Data included in the article/referenced in the report.

## Abbreviations

3' UTR: 3' Untranslated region
AD: Alzheimer's disease
ATP: Adenosine triphosphates
BCL2: B-Cell Lymphoma 2
CD4⁺: Cluster of Differentiation 4 Positive T-Cells
CDR1as: Cerebellar Degeneration-related Protein 1 Antisense RNA
circRNA: CircRNA
circDLGAP4: CircRNA DLG-associated Protein 4
circDLGAP4: Circular RNA Disc Large Associated Protein 4
circSNCA: CircRNA Derived from Alpha-synuclein
circZIP2: CircRNA Zinc Finger Protein 2
circIGFB2: CircRNA insulin-like growth factor binding protein 2
circHDGFRP3: CircRNA derived from the Hepatoma-Derived Growth Factor-Related Protein 3 gene
CNS: Central nervous system
COX: Cytochrome c oxidase
DJ-1 (PARK7): Protein deglycase DJ-1
D-loop: Displacement loop
ETC: Electron transport chain
FUNDC: FUN14 domain-containing protein
GAS5: Growth arrest-specific 5
HD: Huntington's disease
HMC3: Human microglial cells
HOTAIR: HOX transcript antisense intergenic RNA
lncRNA: Long noncoding RNA
LAMP2A: Lysosome-associated membrane protein 2A
LRRK2: Leucine-Rich Repeat Kinase 2
LT: Lymphotoxin
MALAT1: Metastasis-associated lung adenocarcinoma transcript 1
MAPK14: Mitogen-activated protein kinase 14
MEF2A: Myocyte enhancer factor 2A
miRNA (miR): MicroRNA
mETC: Mitochondrial electron transport chain
MMP: Mitochondrial membrane potential
MRncRNAs: Mitochondrial-related non-coding RNAs
mtDNA: Mitochondrial heat shock protein 70
ncRNA: Noncoding RNA
NDs: Neurodegenerative diseases
nDNA: Nuclear DNA
NEAT1: Nuclear paraspeckle assembly transcript 1
NF-κB: Nuclear factor kappa-light-chain-enhancer of activated B cells
NIC: NIP3-Like Protein X
NORAD: Non-coding RNA activated by DNA damage
NRF1/2: Nuclear factor erythroid 2-related Factor ½
NUMTs: Nuclear mitochondrial DNA sequences
OPCs: Oligodendrocyte precursor cells
OxPhos: Oxidative Phosphorylation
PARK2: Parkin
PBMCs: Peripheral blood mononuclear cells
PD: Parkinson's disease
PGC-1α: Peroxisome proliferator-activated receptor gamma coactivator 1-Alpha
PINK1: PTEN-Induced Kinase 1
RBM47-AS1: RNA binding motif protein 47 antisense RNA 1
rRNA: Ribosomal RNA
ROS: Reactive Oxygen Species
RRMS: Relapsing–remitting multiple sclerosis
SIRT1/4: Sirtuin ¼
SNCA: Alpha-Synuclein
SOCS-1/3: Suppressor of cytokine signaling 1/3
SPMS: Secondary progressive multiple sclerosis
TNFAIP1: Tumor necrosis factor alpha-induced protein 1
TFAM: Mitochondrial transcription factor A
TIM: Translocase of the inner membrane
TOM: Translocase of the outer membrane
TNFα: Tumor necrosis factor alpha
VDAC: Voltage-dependent anion channel
vGAT: Vesicular GABA transporter
